# Symptoms and risk factors of *Cryptosporidium hominis* infection in children: data from a large waterborne outbreak in Sweden

**DOI:** 10.1007/s00436-017-5558-z

**Published:** 2017-08-04

**Authors:** Sara Adler, Micael Widerström, Johan Lindh, Mikael Lilja

**Affiliations:** 1 Department of Public Health and Clinical Medicine, Unit of Clinical Research Centre–Östersund, Umeå University, Umeå, Sweden; 2 Department of Clinical Microbiology, Unit of Communicable Disease Control and Prevention–Östersund, Umeå University, Umeå, Sweden; 3 Department of Cell and Molecular Biology, BMC, Uppsala University, Uppsala, Sweden

**Keywords:** Cryptosporidiosis, Children, Risk factors, Symptoms

## Abstract

*Cryptosporidium* is a major cause of diarrheal disease worldwide. In developing countries, this infection is endemic and in children, associated with growth faltering and cognitive function deficits, with the most severe impact on those aged <2 years. Little has been reported about symptoms and risk factors for children in industrialized countries, although the disease incidence is increasing in such regions. In November 2010, a large waterborne outbreak of *C. hominis* occurred in the city of Östersund in Sweden. Approximately 27,000 of the 60,000 inhabitants were symptomatic. We aimed to describe duration of symptoms and the risk factors for infection with *C. hominis* in children aged <15 years in a Western setting*.* Within 2 months after a boil water advisory, a questionnaire was sent to randomly selected inhabitants of all ages, including 753 children aged <15 years. Those with ≥3 loose stools/day were defined as cases of diarrhoea. The response rate was 70.3%, and 211 children (39.9%) fulfilled the case definition. Mean duration of diarrhoea was 7.5 days (median 6, range 1–80 days). Recurrence, defined as a new episode of diarrhoea after ≥2 days of normal stools, occurred in 52.5% of the cases. Significant risk factors for infection, besides living within the distribution area of the contaminated water plant, included a high level of water consumption, male sex, and a previous history of loose stools. The outbreak was characterized by high attack and recurrence rates, emphasizing the necessity of water surveillance to prevent future outbreaks.

## Introduction

Globally, *Cryptosporidium* accounts for a majority of waterborne outbreaks of protozoan parasitic diseases (Baldursson and Karanis 2011; Checkley et al. 2015; Efstratiou et al. 2017; Karanis et al. 2007). Cryptosporidiosis is self-limiting in individuals who are immunocompetent but can be very severe in those who are immunocompromised, and this illness most often affects children, especially those <2 years of age (Caccio and Chalmers 2016). To date, 31 species of *Cryptosporidium* have been identified, and *C. hominis* and *C. parvum* account for >90% of human infections (Chalmers et al. 2011; Zahedi et al. 2016). The route of transmission is faecal–oral via ingestion of oocysts present primarily in contaminated water or food, although direct transmission can also occur through direct physical contact with an infected person or animal or inhalation of oocysts (Bouzid et al. 2013; Karanis et al. 2007; Mor et al. 2010).

Once ingested, an oocyst releases sporozoites that penetrate and infect the host’s intestinal epithelial cells, leading to diarrhoea, abdominal pain, nausea, and vomiting. The severity of cryptosporidiosis depends chiefly on factors associated with the host, particularly the individual’s immune status. The incubation period is normally 2–10 days (average 7 days), and the symptoms of acute gastroenteritis last approximately 2 days to 3 weeks in adults (Cama et al. 2008; Frisby et al. 1997; Insulander et al. 2005; Tangermann et al. 1991; Widerstrom et al. 2014; Yamamoto et al. 2000). The acute symptoms are equivalent in patients infected with *C. hominis* and those with *C. parvum*, although more variable duration of the symptoms has been described in the former group (Chalmers and Davies 2010). Indeed, some individuals infected with *C. hominis* experience more prolonged complaints, including sequelae of extraintestinal symptoms such as joint pain, eye pain, and fatigue, although these occur less often in children (Hunter et al. 2004; Rehn et al. 2015). Shedding of oocysts usually ends within a week after cessation of diarrhoea but can continue for several weeks (Chalmers and Davies 2010).

Most studies of children with cryptosporidiosis have been performed in developing countries, where diarrhoea due to this illness is a major concern (Kotloff et al. 2013), and the infection is known to be associated with cognitive function deficits and growth faltering and stunting (Ajjampur et al. 2010). Re-infections frequently occur; that is, children suffer multiple infections with the same or different *Cryptosporidium* spp. or with some other genotype (Ajjampur et al. 2010; Cama et al. 2008). Recurrence of diarrhoea within a week after the end of a previous episode has also been described (MacKenzie et al. 1995; Newman et al. 1999). Several studies in developing countries have confirmed that young age is a risk factor for acquiring cryptosporidiosis (Kotloff et al. 2013; MacKenzie et al. 1995), although the severity of symptoms and the incidence of infections decrease with age, possibly due to the development of immunity (Ajjampur et al. 2010; Bouzid et al. 2013). Other risk factors described in such countries are low birth weight and male sex, whereas breast-feeding has been reported to offer protection (Creek et al. 2010; Molbak et al. 1994; Newman et al. 1999).

Considering Western countries, the literature offers little information regarding risk factors and symptoms in children. One investigation of a large outbreak in Milwaukee, Wisconsin, in the USA identified immune suppression, HIV, and age ≥1 year as risk factors (Cicirello et al. 1997). Also, a study in London, England, showed a higher rate of chronic diarrhoea following *Cryptosporidium* infection in children ≤2 years of age (Phillips et al. 1992).

*Cryptosporidium* oocysts frequently occur in surface water. In Sweden in 2011, *Cryptosporidium* oocysts were found in 11.5% of analysed samples of raw water, which is equivalent to levels that have been observed in other countries (Chalmers et al. 2010; Robertson and Gjerde 2001). Clearly, to prevent waterborne outbreaks, it is essential to monitor the quality of both raw water and drinking water and to evaluate the efficiency of current barriers in water treatment plants (DeSilva et al. 2016). Also of importance in this context is that a rising number of outbreaks associated with recreational water facilities have been reported (Yoder and Beach 2010), and it is expected that climate changes will further increase the number of outbreaks in the future (Young et al. 2015).

In November 2010, an extensive waterborne outbreak of *Cryptosporidium* occurred in the city of Östersund, which is located in central Sweden and has a population of 60,000. An estimated 27,000 inhabitants were symptomatic, thus representing the largest outbreak in Europe to date. Oocysts were detected in raw water from Lake Storsjön, in drinking water from the waterworks that supplied approximately 85% of the inhabitants of the city, and in faecal samples from sick patients. All of the analysed samples contained the *C. hominis* subtype IbA10G2. The number of individuals with gastrointestinal symptoms increased from mid-November, and a boil water advisory was issued on November 26; thereafter, the number of new cases rapidly declined. On 23 December, a UV water disinfection system was installed as a complement to the previous standard purification using ozonation and chloramination, and the boil water advisory was cancelled on 18 February 2011 (Widerstrom et al. 2014).

The aim of this study was to describe the duration of symptoms of *C. hominis* infection and the risk factors for such illness in children aged <15 years in a Western setting*.*

## Materials and methods

A retrospective study was conducted within 2 months after the boil water advisory was issued. One thousand five hundred eight randomly selected inhabitants of all ages living in the Östersund municipality were sent a questionnaire concerning the following: onset and occurrence of possible symptoms of cryptosporidiosis and possible risk factors, such as medical history, number of family members infected, and how many glasses of water that were consumed daily. Parents were asked to respond on behalf of children <15 years of age. A reminder was sent to those who had not responded within 2 weeks. The methodology has previously been described in detail (Widerstrom et al. 2014). Concurrently, the same questionnaire was sent to an additional 600 randomly selected children (i.e. subjects that were not included in the cited study) who were aged 0–5 years and lived in Östersund municipality. In total, 753 children aged <15 years received the questionnaire. Information about the distribution of water from the waterworks to the respondents’ homes was ascertained through population registers.

We used the following definition of diarrhoea set by the World Health Organization (WHO): ≥3 loose or liquid stools/day (WHO 2016). Children aged <15 years with onset of diarrhoea after 1 November 2010 and before 31 January 2011 were defined as cases. A relapse was defined as recurrent diarrhoea after ≥2 days of normal stools.

Statistical analyses were performed using SPSS (version 19, SPSS Inc. Chicago, IL, USA). Logistic regression was applied to investigate relationship between risk factors and clinical cryptosporidiosis as the outcome variable. Independent samples *t* test was used to evaluate differences between age and sex groups. All analyses of age-related differences among children were performed using the following four age groups: <2, 2–5.99, 6–9.99, and 10–14.99 years. Probabilities of <0.05 were considered statistically significant. Six respondents that stated unlikely high water consumption (i.e. >30 glasses of water/day) were excluded from analyses regarding water consumption. Some of the risk factors that were analysed in the adult population included in our previous study (i.e. irritable bowel syndrome, inflammatory bowel disease, diabetes, cortisone medication, rheumatic disease, and peptic ulcer medication) (Widerstrom et al. 2014) were not assessed in children in the present investigation due to too few positive answers.

## Results

The overall questionnaire response rate was 70.3%, and male and female children of all ages responded at equivalent rates. The proportion of children aged 0–5 years was 79.7%, and 39.9% (211/529) of all the children (more males than females: 45.1 vs. 34.5%, *P* = 0.015) met the case definition (Table 1). The attack rate was 43.6% for children who lived in the parts of Östersund served by the contaminated water treatment plant compared to 25.7% for children who lived in areas that were not served by that plant (*P* = 0.001). Most cases (>50%) were detected within ±2 weeks of the date the boil water advisory was issued. Thereafter, the number of cases rapidly declined, but symptoms appeared in January 2011 in 11.0% of the cases. A median of three individuals living in the same household as a case exhibited gastrointestinal symptoms, and the corresponding number for those living together with non-cases was one. A small percentage (0.4%) reported that they did not follow the boil water advisory, and 7.6% indicated that they occasionally failed to do so.

**Table 1:** Baseline characteristics

	*n* (*N*)	Fulfilling case definition (%)	*P* between groups
Total	529 (753)	39.9	
Sex			0.015
Female	261 (373)	34.5	
Male	268 (380)	45.1	
Age (years)			NS
<2	145 (199)	42.8	
2–5.99	280 (401)	38.6	
6–9.99	51 (77)	39.2	
10–14.99	53 (76)	39.6	
Lived within area served by WTP-Ö	420 (603)	43.6	0.001
Lived outside area served by WTP-Ö	109 (150)	25.7	

The number of responders (*n*), the total number of questionnaires (*N*) distributed, and the percentage fulfilling the case definition stratified by sex, age, and residency within or outside the area served by the contaminated water plant; *Case* defined as onset of ≥3 loose stools daily, from 1 November 2010 through 31 January 2011; *WTP-Ö* designates the contaminated water treatment plant in Östersund

*NS* not significant

### Symptoms

The most commonly presented symptoms are listed in Table 2. Watery diarrhoea was reported by 82.0% of cases but also by 6.5% of non-cases. Among cases, two symptoms were more common in female children: headache (51 vs. 28% in males, *P* = 0.03) and abdominal pain (76 vs. 61.1% in males, *P* = 0.035). The mean duration of diarrhoea was 7.5 days (median 6, range 1–80 days), and no differences in duration were found between male and female children or between different age groups (Table 3). However, the duration of diarrhoea was longer for some children: 13.2% reported ≥14 days, and 2.0% reported ≥30 days. Also, 33.5% of the participants reported one relapse, 11.2% reported two relapses, and 7.8% indicated ≥3 relapses; thus, no relapses were reported by 47.6% of the respondents. There was no difference in recurrence rate between the sexes or between age groups.

**Table 2:** Clinical symptoms in case and non-case participants

	Case		Non-case		*P*
	*n*	%	*n*	%	
Watery diarrhoea	152	82.0	18	6.5	<0.0005
Abdominal pain	128	67.4	34	12.1	<0.0005
Fatigue	129	66.8	27	10.1	<0.0005
Nausea	80	47.9	20	7.1	<0.0005
Fever	82	44.3	12	4.4	<0.0005
Vomiting	75	42.4	29	10.4	<0.0005
Headache	63	38.7	14	5.2	<0.0005
Eye pain	13	8.6	5	1.9	0.001
Joint ache	6	4.0	4	1.5	NS
Bloody diarrhoea	2	1.3	0	0.0	NS

*Case* defined as onset of ≥3 loose stools daily from 1 November 2010 through 31 January 2011

*NS* not significant

**Table 3:** Days with diarrhoea in cases stratified by sex and age

	Median (days)	Mean (days)	SD	*P*
All	6	7.5	8.0	
Sex				NS
Male	6.5	7.9	7.3	
Female	5	7.1	8.5	
Age (years)				NS
<2	7	8.1	6.2	
2–5.99	5	6.6	5.3	
6–9.99	6.5	12.6	19.2	
10–14.99	5	5.1	2.7	

*Case* defined as onset of ≥3 loose stools daily, from 1 November 2010 through 31 January 2011

*NS* not significant

### Risk factors and underlying chronic conditions

Table 4 presents risk factors for clinical cryptosporidiosis. Male sex and also high consumption of water increased the risk. Male and female children consumed the same amount of water (4.7 glasses/day). The youngest children aged 0–1 years consumed less water than those aged 2–5 years (mean 3.7 vs. 4.4 glasses/day, *P* = 0.003).

**Table 4:** Risk factors for cryptosporidiosis

	Univariate model			Multivariate model		
	OR	95% CI	*P*	OR	95% CI	*P*
Glasses of water/day						
<2	1	ref.		1	ref.	
2–5	2.8	(1.3–6.1)	0.009	2.9	(1.3–6.3)	0.009
≥5	3.6	(1.6–8.2)	0.002	3.4	(1.5–8.0)	0.005
Sex						
Female	1	ref.		1	ref.	
Male	1.6	(1.1–2.2)	0.015	1.6	(1.1–2.4)	0.013
Living within WTP-Ö-served area	2.3	(1.4–3.7)	0.001	2.3	(1.4–2.9)	0.001
Previous loose stool	2.6	(1.4–4.8)	0.003	2.7	(1.4–5.1)	0.004
Previous abdominal pain	2.1	(1.1–4.2)	0.03	NS		
Previous “bubbly” stomach	1.1	(0.4–2.7)	NS			
Cow milk allergy	1.2	(0.5–3.1)	NS			
Lactose intolerance	0.7	(0.3–1.8)	NS			

Values given are odds ratios (ORs) with 95% confidence intervals (CIs); all statistically significant factors in the univariate model were included in the multivariate model; the subheading *Glasses of water* refers to the number of glasses of unboiled tap water (including infant formula) that were consumed daily; *WTP-Ö* designates the contaminated water treatment plant in Östersund

*NS* not significant, *ref*. reference

## Discussion

To our knowledge, this is the most extensive study describing risk factors and clinical symptoms among children aged <15 years with cryptosporidiosis in a Western setting. In particular, this investigation focused on the youngest children aged 0–5 years, who constituted the largest group in the evaluation. We found no evidence that young age was a significant risk factor for cryptosporidiosis, as is often found in studies conducted in developing countries (Kotloff et al. 2013; Molbak et al. 1994). However, male children were at significantly higher risk of cryptosporidiosis, which concurs with previous reports (Agnew et al. 1998; Molbak et al. 1994) and with increasing data showing that morbidity from various infectious diseases occurs more frequently in male than that in female children (Muenchhoff and Goulder 2014). The general findings of our investigation with regard to symptoms and the duration of diarrhoea are also consistent with previous studies (Cicirello et al. 1997; Phillips et al. 1992).

No differences between age groups were found with respect to incidence, symptoms, or duration of diarrhoea, which might be partly explained by nutritional factors, considering that cryptosporidiosis has been observed to be more prevalent in malnourished children (Bouzid et al. 2013; Caccio and Chalmers 2016). Most children in Östersund were probably exposed to *Cryptosporidium* for the first time during the noted outbreak, whereas older children in countries where *Cryptosporidium* is endemic may develop partial immunity over time (Checkley et al. 2015). In our study, more water was consumed by the older children than those who were youngest, which might have resulted in ingestion of a larger number of oocysts. Oocyst density in the drinking water was relatively low during the outbreak in Östersund, although this was measured after the boil water advisory had been issued (Widerstrom et al. 2014). Notably, previous reports have described a protective impact of breast-feeding (Creek et al. 2010; Molbak et al. 1994), an aspect that might also have contributed to a lower incidence of infection among the youngest children in our study.

Although >90% of the questionnaire respondents followed the boil water advisory, new cases of cryptosporidiosis occurred in January. Symptoms were reported by more individuals in households with cases than in households with non-cases, possibly indicating the presence of secondary transmission or potentially the habit of consuming large amounts of drinking water within such household. Secondary transmission among children was suggested to be an important transmission route in several outbreaks (Heijbel et al. 1987; Johansen et al. 2015), but it is also possible that some of the affected children in those studies drank tap water without the parents’ knowledge. In addition to this, asymptomatic carriage is prevalent (Ajjampur et al. 2010; Collinet-Adler and Ward 2010; Cordell et al. 1997; Davies and Chalmers 2009); for example, only 78% of the children found to be positive for *Cryptosporidium* in Milwaukee presented with diarrhoea (Cicirello et al. 1997).

A high rate of recurrence of diarrhoea in individuals affected by cryptosporidiosis as reported by Hunter et al. (2004) is consistent with the recurrence rates observed in adults in both Östersund (Widerstrom et al. 2014) and Milwaukee (MacKenzie et al. 1995). Whether recurrence in diarrhoea in the present subjects was due to autoinfection or new secondary transmission is not possible to determine based on our data. It is well documented that oocyst shedding continues after cessation of symptoms (Chalmers and Davies 2010), potentially representing a risk that individuals will re-infect themselves or others. In Östersund, it is unlikely that recurring diarrhoea could have been due to other pathogens, because no laboratory-confirmed cases during the outbreak in that city were positive for any gastrointestinal pathogens other than *Cryptosporidium* (Widerstrom et al. 2014).

The WHO definition of persistent diarrhoea (i.e. lasting ≥14 days) (WHO 2016) was fulfilled by 13.2% of cases in our investigation, and a high rate of persistent diarrhoea among children with cryptosporidiosis was described in a previous study conducted in London, England (Phillips et al. 1992). In Östersund, only 2.0% met the WHO definition of chronic diarrhoea (i.e. lasting ≥30 days), although this may be an underestimation, because the follow-up of diarrhoea ended in February 2011.

Strengths of our investigation include a large study population and a high response rate only a short time after a *C. hominis* outbreak. A major weakness is that cases may have been misclassified. In short, it is possible that some cases of diarrhoea were not caused by *C. hominis*, considering that many were not verified by analysis of faecal samples. On the other hand, a number of non-cases also presented with gastrointestinal symptoms, indicating that some of them might also have been infected with *Cryptosporidium*.

In conclusion, the outbreak in Östersund was characterized by a high attack rate and a high rate of recurrent diarrhoea that led to many days with symptoms in a large number of the investigated children. In addition to living within the distribution area of the contaminated water plant, other significant risk factors included consumption of large amounts of water, male sex, and a previous history of loose stools. This study underlines that employing insufficient drinking water control measures can have a pronounced impact on public health.
